# Combined effect of microbially derived cecal SCFA and host genetics on feed efficiency in broiler chickens

**DOI:** 10.1186/s40168-023-01627-6

**Published:** 2023-09-01

**Authors:** Zhengxiao He, Ranran Liu, Mengjie Wang, Qiao Wang, Jumei Zheng, Jiqiang Ding, Jie Wen, Alan G. Fahey, Guiping Zhao

**Affiliations:** 1 State Key Laboratory of Animal Nutrition; Key Laboratory of Animal (Poultry) Genetics Breeding and Reproduction, Ministry of Agriculture, Institute of Animal Sciences, Chinese Academy of Agricultural Sciences, Beijing, 100193 China; 2 School of Agriculture and Food Science, University College Dublin, Dublin, Ireland

**Keywords:** Feed efficiency, Cecal microbiota, Genetic variations, SCFAs, Broiler

## Abstract

**Background:**

Improving feed efficiency is the most important goal for modern animal production. The regulatory mechanisms of controlling feed efficiency traits are extremely complex and include the functions related to host genetics and gut microbiota. Short-chain fatty acids (SCFAs), as significant metabolites of microbiota, could be used to refine the combined effect of host genetics and gut microbiota. However, the association of SCFAs with the gut microbiota and host genetics for regulating feed efficiency is far from understood.

**Results:**

In this study, 464 broilers were housed for RFI measuring and examining the host genome sequence. And 300 broilers were examined for cecal microbial data and SCFA concentration. Genome-wide association studies (GWAS) showed that four out of seven SCFAs had significant associations with genome variants. One locus (chr4: 29414391–29417189), located near or inside the genes *MAML3*,* SETD7*, and* MGST2*, was significantly associated with propionate and had a modest effect on feed efficiency traits and the microbiota. The genetic effect of the top SNP explained 8.43% variance of propionate. Individuals with genotype AA had significantly different propionate concentrations (0.074 vs. 0.131 μg/mg), feed efficiency (FCR: 1.658 vs. 1.685), and relative abundance of 14 taxa compared to those with the GG genotype. *Christensenellaceae* and *Christensenellaceae_R-7_group* were associated with feed efficiency, propionate concentration, the top SNP genotypes, and lipid metabolism. Individuals with a higher cecal abundance of these taxa showed better feed efficiency and lower concentrations of caecal SCFAs.

**Conclusion:**

Our study provides strong evidence of the pathway that host genome variants affect the cecal SCFA by influencing caecal microbiota and then regulating feed efficiency. The cecal taxa *Christensenellaceae* and *Christensenellaceae_R-7_group* were identified as representative taxa contributing to the combined effect of host genetics and SCFAs on chicken feed efficiency. These findings provided strong evidence of the combined effect of host genetics and gut microbial SCFAs in regulating feed efficiency traits.

Video Abstract

**Supplementary Information:**

The online version contains supplementary material available at 10.1186/s40168-023-01627-6.

## Introduction

Feed is one of the most expensive components of the farm animal industry costs, accounting for up to 70% of production costs [1]. Strategies to improve production without additional feed supplies are vital to ensuring the profitability and sustainability of the industry. Feed efficiency (FE) depends on the relation between the feed intake (FI) and the growth (or bodyweight gain) of an animal and is described by several indexes, such as feed conversion ratio (FCR) and residual feed intake (RFI). Feed efficiency is influenced by several factors, including the breed of the birds and their sex, age, diet, and management [2]. Energy intake and consumption are the basic daily biological functions of chickens, in theory, the ability to derive more energy from the same amount of feed and reduce all energy consumption apart from that required for daily maintenance would likely reduce feed intake [3, 4]. Thus, feed efficiency could be regulated by energy metabolism and feeding behaviors such as appetite [5, 6]. FCR and RFI are two indicators commonly used to evaluate the feed efficiency of livestock [7, 8]. RFI is preferred over FCR since it reflects the variation in the efficiency of feed utilization by broilers, which is independent of growth traits [9]. The heritability of RFI was reported to be between 0.23 and 0.49, and many genome-wide association studies (GWAS) have indicated that RFI is associated with host genome variation [10–15]. Over the last 50 years, the feed efficiency of commercial breeds improved by 50% due to quantitative genetic selection [16]. Additionally, the gut microbiota can markedly affect animal feed efficiency, as symbionts influence host metabolism [17].

The chicken gastrointestinal tract (GIT) includes compartments with varied physiological roles and environments that drive the spatial distribution of microbial populations [18]. Lower species richness in the intestine of chickens is accompanied by greater feeding efficiency, but this difference is not reflected in fecal samples [19, 20]. However, several studies found that bacterial diversity within the intestinal tract is higher in birds with lower FCR or higher feed efficiency [21–25]. Because the cecum is the primary site for food fermentation in monogastric animals, many cecal microbiota studies have been conducted over a wide range of microbiota diversity. Several studies have attempted to identify the intestinal microbes associated with RFI in broiler and layer chickens [15, 22, 26–28]. Nevertheless, findings to date have been inconsistent and sometimes contradictory. The low repeatability of microbial trials might be due to the susceptibility of intestinal microbiota communities to differences in diet, environment, management, age, and breed [29]. Many studies calculated the heritability of microbiota, showing a low average of 0.068 [30]. Furthermore, there is a broad range of microbial taxa in the environment [31], which increases the complexity of microbial studies.

A previous study indicated that short-chain fatty acids (SCFAs) present in the caecum were of microbial origin in a germ-free study [32]. SCFAs are well known as energy sources [33]. Hence, identifying a more energy-efficient microbiota is necessary to develop effective strategies to improve feed utilization. There is no previous study on the association between the host genome and SCFA production. As a previous study reported, SCFAs are produced by the gut microbiota, and the interactions between the host genome and the microbiota were reported [15]. SCFAs can work as signaling molecules with the help of G protein-coupled receptors (GPCRs), which are called free fatty acid receptors (FFARs) [33]. GPR43/FFAR2 and GPR41/FFAR3 can interact with the major SCFAs (acetate, propionate, and butyrate), which regulate energy expenditure, preadipocyte differentiation, and appetite control [34, 35]. Feed efficiency traits have been widely investigated in cattle and are affected by feeding behavior and energy metabolism, which could be related to SCFA metabolism due to their effects on appetite and energy homeostasis [5, 36, 37]. Previous RNA-seq results in divergent RFI groups found that differentially expressed genes, such as *CAMP*, *LPL*,* PCK1*, and *CCKAR* interact with GPCRs in lipid and energy metabolism [38–41]. Hence, there is some evidence that the gut microbiota could produce SCFAs and possibly regulate host feed efficiency through energy- and appetite-related pathways.

**Fig. 1:**
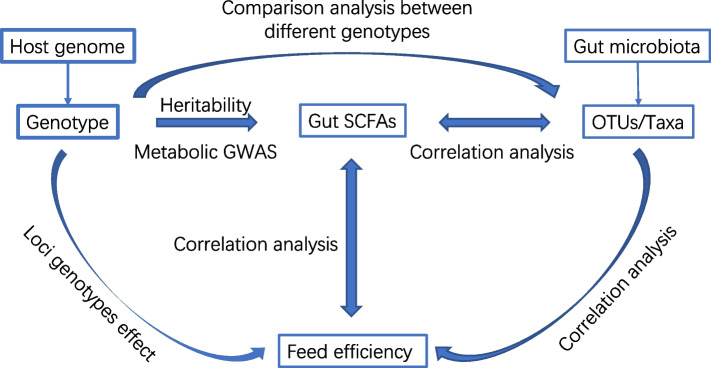
The overall workflow. The workflow works on the pathway “host genome variants-cecal microbiota-cecal SCFA-feed efficiency.” The GWAS study was conducted on gut SCFAs and feed efficiency. The heritability of gut SCFAs was estimated based on genomic data. The SCFA-related SNPs were used to test the loci effect on the gut microbiota and feed efficiency to help screen the vital components of the microbiota. The correlation tests were conducted among gut SCFAs, microbial taxa, and feed efficiency traits

The cecum is the primary fermentation site in poultry and the major site of microbial SCFA production. Changes in the microbiota and SCFA production can affect feed efficiency. Therefore, assessing the relationship between host genetics and the gut microbiota, cecal SCFAs and feed efficiency will improve our understanding of the potential biological variations in feed efficiency and design sustainable approaches to improve feed efficiency in chickens. To achieve this goal, data of host genomics, microbial taxa, and SCFAs in cecum segments were used to clarify the relationships among the microbiota, cecal SCFAs, and host genetic variation. The overall workflow of the present analyses is shown in Fig. 1.

## Materials and methods

### Animals

All chickens were obtained from the fast-growing white-feathered pure line, produced by Xinguang Agricultural and Animal Industrials Co., Ltd. (Mile, China). This line was selected for eight generations for high body weight and feed efficiency traits. RFI testing was conducted on a total of 464 broilers. They were housed in identical individual cages (length × width × height, 30 × 25 × 45 cm) and fed ad libitum. Each day, the amount of fresh feed provided was recorded individually, and residual feed was recorded daily and removed during the period from 28 to 40 days of age. During this period, the animals were fed a corn-soybean meal diet, and detailed information about the diet is described in Additional file 1: Table S1. The bodyweight of each chicken at 28 and 40 days of age was measured using an electronic scale. The RFI calculation method was described by Li et al. [13]. The descriptive statistics of these phenotypes are summarized in Additional file 2: Table S2. The correlation coefficient between RFI and ADFI was 0.61, and significant correlations were found in coefficients between RFI and FCR (0.79), the ratio of the breast (− 0.23), and abdominal fat ratio (0.37) (Additional file 3: Figure S1).

At the age of 41 days, the whole blood was collected from each bird from the wing vein using a vacuum blood tube. Furthermore, broilers were sacrificed 2 h after the last feed to allow time for the feed to be digested in the GIT. Each bird was then euthanized by cervical dislocation. The abdominal fat tissue, whole breast muscle, and thigh on the right side were carefully dissected and weighed promptly with an electronic balance (0.1 g precision). Moreover, the cecal contents (including chyme and mucosa) were collected immediately. All the samples were snap-frozen in liquid nitrogen, transported to the laboratory and stored at − 80 °C for subsequent studies.

### Genotyping and quality control

Genomic DNA was extracted from blood samples with the phenol–chloroform method [42]. In total, 464 broilers were resequenced with 150 bp paired-end reads on an Illumina NovaSeq 6000 platform with an average depth of approximately 10 × 1 L coverage conducted by Beijing Compass Biotechnology Co., Ltd. (Beijing, China). Variant calling was performed according to a standardized bioinformatics pipeline for all samples [43, 44]. Specifically, clean sequencing data were aligned to the chicken reference genome (GRCg6a/galGal6: https://ftp.ncbi.nlm.nih.gov/genomes/all/GCF/000/002/315/GCF_000002315.6_GRCg6a/) with the Burrows-Wheeler Aligner (BWA)-MEM algorithm [45]. Then, PCR duplicates were removed, and local indel realignment and base quality score recalibration were performed with the Genome Analysis Toolkit (GATK version 3.5) [46]. Variant calling was performed via HaplotypeCaller in GVCF mode with joint genotyping on all samples. Finally, SNPs were filtered with the GATK VariantFiltration protocol. The filtering settings were as follows: variant confidence score (QUAL) < 30.0, QualByDepth (QD) < 2.0, ReadPosRankSum <  − 8.0, total depth of coverage (DP) < 4.0, and FisherStrand (FS) > 60.0. In addition, quality control of the reference panel was conducted with the criteria of MAF ≥ 0.05, only bi-allelic sites, genotyping missing < 0.2, mean depth value between 3 and 30, and site quality value higher than 30. After filtering, 9,540,946 autosome variants remained for the 464 sequenced birds, LD decay was conducted by PopLDdecay [47], and the average LD level in a 5-kb interval was 0.17 (Additional file 4: Figure S2).

### 16S rRNA gene sequencing and analysis

Three hundred cecal samples were used to conduct 16S rRNA amplicon sequencing. The total DNA of cecal contents was extracted by a QIAamp DNA Stool Mini Kit (QIAGEN, Hilden, Germany). Eight cecal samples were excluded because of DNA extraction failure. Finally, 292 microbial DNA samples were used for 16S rRNA sequencing. Two divergent RFI groups were divided by the rank of the RFI value only. Sixty-nine top-ranked and sixty-nine bottom-ranked RFI samples were assigned to high and low groups. The V4 region of the 16S rRNA gene was amplified using the primer pair 515F/806R (5′-GTGCCAGCMGCCGCGGTAA-3′ and 5′-GGACTACHVGGGTWTCTAAT-3′), and the amplicons were purified and quantified using Agencourt AMPure Beads and the PicoGreen dsDNA Assay Kit (Invitrogen, Carlsbad, CA, USA), respectively. After quantification, the barcoded V4 amplicons were pooled and subsequently sequenced using an Illumina MiSeq platform (Illumina, San Diego, CA) to generate 300 bp paired-end reads at Shenzhen BGI Technology Services Co., Ltd. For each sample, there were approximately 50,000 clean reads. Amplicon sequencing bioinformatics was performed with EasyAmplicon v1.0 [48]. Paired-end sequence data were merged, quality-filtered, and dereplicated using VSEARCH v2.15 subcommand –fastq_mergepairs, –fastx_filter, and –derep_fulllength, respectively [49]. Then, the non-redundancy sequences were denoised into amplicon sequence variants (ASVs) with USEARCH v10.0 [50] (via -cluster_otus or unoise3). Chimeras were removed by VSEARCH –uchime_ref against the SILVA database [51]. Feature tables were created by vsearch –usearch_global. The taxonomy of the features was classified by the USEARCH sintax algorithm in SILVA v123. Diversity analysis was carried out using the vegan v2.5–6 package (https://cran.r-project.org/web/packages/vegan/), and visualized by using the ggplot2 v3.3.2 (https://cran.r-project.org/web/packages/qqman/) package in R v4.0.2. LEfSe was conducted with the online platform ImageGP (http://www.ehbio.com/ImageGP-/index.php/Home/Index/LEFSe.html) [52]. Functional profile prediction of microbial communities was conducted by PICRUSt [53], with the Greengenes as the reference database.

### SCFA concentration determination

Three hundred cecal samples, the same as those used for amplicon sequencing population, were used for seven SCFA concentration determinations, including acetate, propionate, isobutyrate, butyrate, isovalerate, valerate, and hexanoate. Briefly, samples were thawed on ice, and approximately 50 g of the sample was added to 400 µl of saturated sodium chloride solution, and 50 µl 3 mmol of saturated sodium chloride solution of hydrochloric acid was added. Ultrasonic oscillation at low temperatures was conducted for 20 min. Then, 500-µl ether was added, oscillated sufficiently, and extracted for 10 min. Next, the supernatant was centrifuged for 10 min at 12,000 r/min and 4℃. Then, 50 mg of anhydrous sodium sulfate was added into the supernatant and oscillated for 3 min. Finally, the mixture was centrifuged at 4500 r/min and 4℃ for 5 min, and the supernatant was used for analysis. A total of 2 µl of the solution was analyzed by a TRACE1300-TSQ9000 gas chromatography-mass spectrometry (GC–MS) instrument (Thermo Fisher Scientific, Waltham, MA, USA) at Shenzhen BGI Technology Services Co., Ltd. To determine the absolute SCFA concentration, SCFA standards were prepared at different dilutions with ultrapure water. Then, the protocol described above was conducted to generate standard curves for the seven SCFAs.

### Evaluating the effects of host genetics on SCFAs and growth performance

The GWAS for SCFA concentrations were performed for 300 individuals, and growth performance traits were performed for 464 individuals, directly using the univariate linear mixed model (LMM) implemented in GEMMA version 0.98.1 software (https://github.com/genetics-statistics/GEMMA/releases) [54]. The SCFA concentrations were log-2 transformed to make them follow a normal distribution (Additional files 5 and 6: Figure S3 and S4). SNP-based heritability analysis was implemented in GCTA (ver 1.93.3) [55]. The GWAS model was described in detail in a previous study [13]. The genotype was dimed as a fixed factor and the additive genetic effect as the random effect. Due to the same generation and sex in this population, no covariate was applied in the LMM model. The statistical model was as follows:

$$y=\alpha +x\beta +u+\epsilon ;u\sim {MVN}_{n}\left(\mathrm{O},{\lambda \tau }^{-1}K\right),\epsilon \sim {MVN}_{n}\left(\mathrm{O},{\tau }^{-1}{I}_{n}\right),$$

where *y* represents the vector of SCFA values, *α* represents the vector of the corresponding coefficients including the intercept, *x* represents the vector of genotypes, *β* represents the effect size of the marker, *u* represents the vector of random polygenic effects, *ϵ* represents the vector of errors, *τ*^*−1*^ represents the variance of the residual errors, *λ* represents the ratio between the two variance components, *K* represents the centered relatedness matrix estimated from 9,540,946 variants, and *I*_*n*_ represents the identity matrix. MVN_*n*_ represents the n-dimensional multivariate normal distribution. The Wald test was used to select SNPs associated with metabolizable efficiency traits.

The genome-wide significance was assessed using the GEC method [56] to infer effective independent tests. A total of 9,540,946 independent tests overall chromosomal SNPs were obtained, and 8,562,703 SNPs were retained. Then, genome-wide significant and suggestive thresholds were set to 5.84 × 10^–9^ (0.05/8,562,703) and 1.17 × 10^–7^ (1/8,562,703), respectively. Manhattan and Q-Q plots were constructed for each trait by the qqman package (https://cran.r-project.org/web/packages/qqman/) in R (version 4.1.0). SNP positions were updated according to the GRCg6a genome version from NCBI. The closest genes to genome-wide significant and suggestive variants were identified using NCBI annotation of the GRCg6a genome version (https://www.ncbi.nlm.nih.gov/data-hub/gene/table/taxon/9031/). The variance in SCFAs explained by SNPs from GWAS results was calculated by the formula described by Shim et al. [57].

### Identification of the specific microbiota association

The associations between qualified taxa, feed efficiency, and SCFA traits were analyzed using a two-part model described by Fu et al. [58]. This model accounts for both binary (present and absent) and quantitative features and is described as follows:

1 $$y=\left\{\begin{array}{l}\beta_1b+e\\\beta_2q+e\end{array}\right.$$

where *y* is the RFI value or SCFA concentration, *b* is a binary feature of a specific microorganism and coded as 0 for absent or 1 for present for each sample, and *q* is the log10-transformed abundance of a specific microorganism. β_1_ and β_2_ are the regression coefficients for the binary and quantitative models, respectively, and *e* is the intercept. The second part of the quantitative analysis was only for the samples in which the specific microorganism was present. The details of the two-part model are illustrated in Additional file 7: Figure S5. *P* values were obtained from the two-part model association analysis and adjusted by the BH method. If the adjusted *P* value from the binary model was less than 0.05, the presence or absence of microorganisms was considered to influence feed efficiency. If the adjusted *P* value from the quantitative model was less than 0.05, feed efficiency was considered associated with the relative abundances of the microorganisms. If the combined *P* value was less than 0.05, feed efficiency was considered to be associated with both the relative abundances of the microorganisms and the presence or absence of microorganisms.

A Spearman correlation analysis between microbiotas and RFI and FCR was conducted to detect specific microorganisms that significantly influenced feed efficiency. A microorganism was considered to have a significant effect if the adjusted *P* values from the two-part model association analysis and Spearman correlation were less than 0.05.

### Statistical analysis and data visualization

The divergent RFI groups were divided by RFI ranking only, the top 69 individuals were allocated to the HRFI group, and the bottom 69 individuals were allocated to the LRFI group. The divergent PA groups followed similar selection criteria, which were only based on PA value ranking. The top 45 PA individuals were allocated to HPA groups, and the bottom 52 PA individuals were allocated to the LPA group. The details of the data describing the divergent groups are shown in Table 1.

**Table 1:** Growth performance description of divergent RFI and PA groups

Population	Num	Bodyweight(g)		ADG	ADFI	FCR	RFI	PA
		28 days	40 days	(g/d)	(g/d)		(g/d)	(μg/mg)
HRFI group	69	1082	2227	95.4	167.46	1.76	8.93	-
LRFI group	69	1087	2210	93.61	148.56	1.59	-8.04	-
Statistical test		*P* = 0.693	*P* = 0.295	*P* = 0.151	*P* = 0.000	*P* = 0.000	*P* = 0.000	-
HPA group	45	1074	2224	95.8	159.69	1.67	0.96	0.127
LPA group	52	1093	2215	93.53	154.67	1.66	− 2.00	0.046
Statistical test		*P* = 0.228	*P* = 0.618	*P* = 0.116	*P* = 0.029	*P* = 0.494	*P* = 0.038	*P* = 0.000

Num. indicates the number of individuals in each group

*ADG* average daily gain, *ADFI* average daily feed intake, *PA* propionate concentration

*P* value indicates the statistical significance of the differences between divergent RFI or PA groups

The comparative analysis was conducted between divergent groups, and the *t* test was conducted by the t.test() function in the R program (version 4.1.0). Welch’s *t* test was used to determine the differences in the pathways/taxon relative abundances with FDR correction in STAMP v2.1.3 [59]. Some scripts about data format are from Microbiome helper [60]. All the pipeline, training materials, and related scripts were deposited in the EasyAmplicon project on GitHub (https://github.com/YongxinLiu/EasyAmplicon2019). The beta-diversity statistic of divergent groups was determined by Adonis in the amplicon package followed by the EasyAmplicon procedure [61]. Differences were considered statistically significant at *P* < 0.05. The plots were generated by the ggplot2 package (https://cran.r-project.org/web/packages/ggplot2/) in the R program.

## Result

### Divergent RFI groups had different microbial communities

**Fig. 2:**
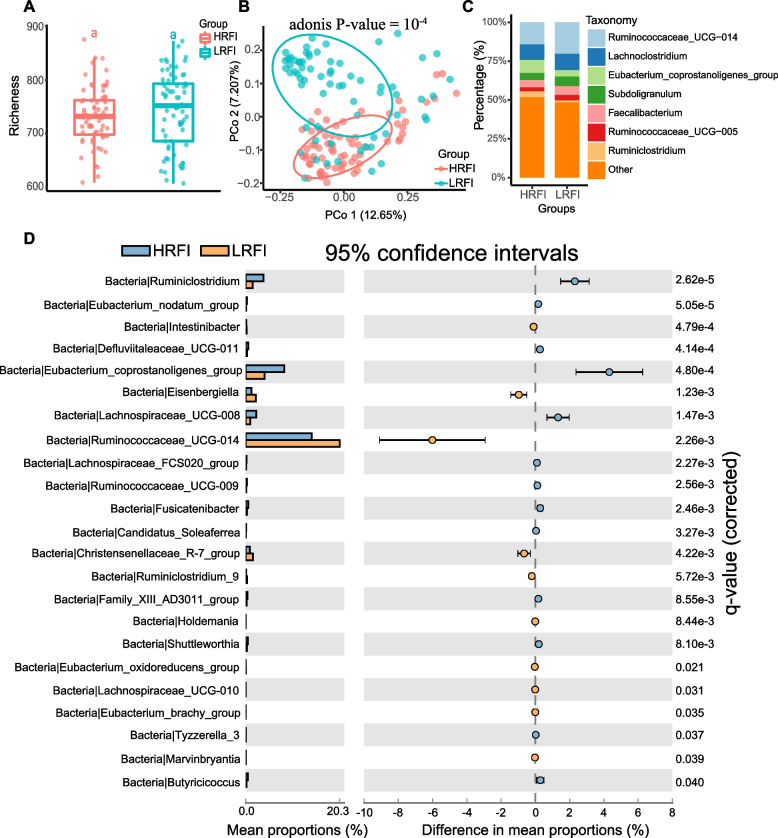
The microbiota composition between the high RFI and low RFI groups. **A** The α-diversity index richness compared between the two groups. **B** The Principal Coordinates Analysis on the ASV level. **C** The cecal microbiota composition of two groups on the genus level. **D** Comparisons of genus components between the two groups

The RFI distributions of the high (HRFI) and low (LRFI) RFI groups followed the normal distribution, respectively (Additional file 8: Figure S6. A). The richness of the high and low RFI groups was almost higher than 700 (Fig. 2A), which represents a sufficient detection rate (Additional file 8: Figure S6. B). The PCoA analysis was conducted by Bray_Curtis distance, and the plot shows that the β-diversities of the high and low groups were significantly different (Fig. 2B). Seven genera were detected as the major composition of chickens in the divergent RFI groups (Fig. 2C). The detected genera accounted for approximately half of the percentage due to the lower detected ratio, and unassigned taxonomies were allocated to the other part. Twenty-three genera were determined to be significant in terms of relative abundance based on the Wilcoxon rank-sum test with an FDR-adjusted *P* value less than 0.05 (Fig. 2D).

### Significant correlations were found between SCFA, microbiota, and feed efficiency traits

**Fig. 3:**
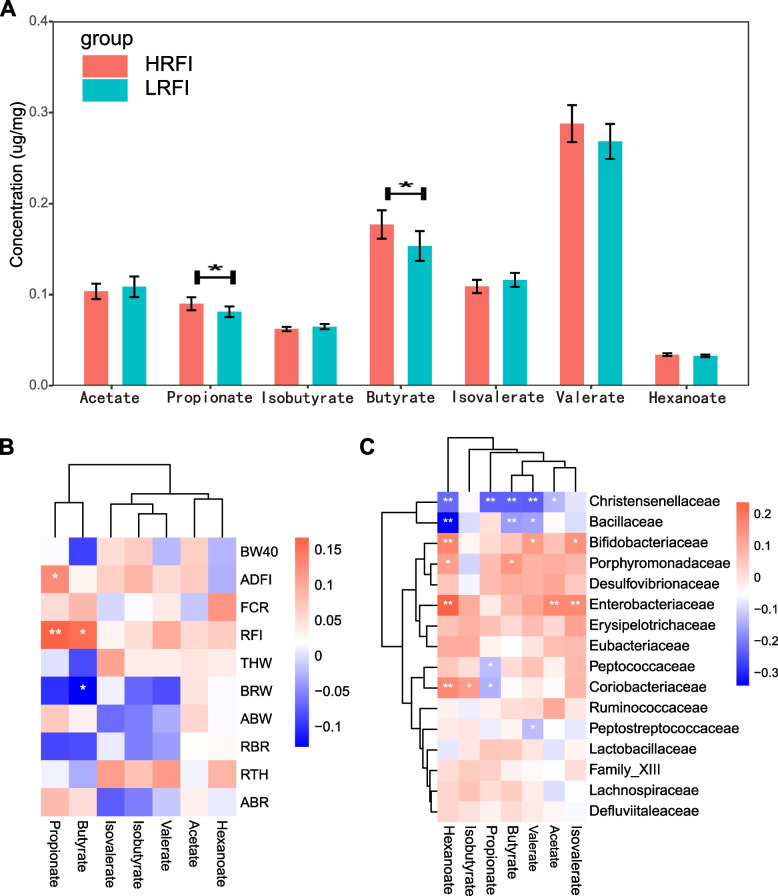
Correlation between SCFAs and feed efficiency and microbial biomarkers of feed efficiency. **A** Bar plots of the concentration of SCFAs among the high and low RFI groups, “*” means there is a statistical difference between divergent RFI groups. **B** Spearman correlation between SCFAs and growth performance. **C** Spearman correlation between SCFAs and taxa at the family level. BW40, body weight at 40 days of age; ADFI, average daily feed intake; THW, thigh weight; BRW, breast weight; ABW, abdominal fat weight; RTH, ratio of thigh weight; RBR, ratio of breast weight; ABR, ratio of abdominal fat

Comparisons of different SCFAs between the high and low RFI groups were conducted (Fig. 3A). The propionate concentration in the HRFI group (0.090) was significantly higher than that in the LRFI group (0.081). A similar trend was also found for butyrate (0.177 vs. 0.153) between HRFI and LRFI groups. Spearman correlations between SCFAs and growth traits were conducted among individuals (Fig. 3B). Propionate and butyrate were positively correlated with RFI but negatively correlated with BRW and RBR. Propionate was positively correlated with ADFI. The correlations between SCFAs and families were calculated. *Christensenellaceae* was negatively correlated with propionate, and *Christensenellaceae* and *Bacillaceae* were negatively correlated with butyrate (Fig. 3C). Only the relative abundance of *Christensenellaceae* significantly differed in divergent RFI groups in the above comparison analysis. Moreover, the heatmap of Spearman correlations between SCFAs and genera can be found in Additional file 9: Figure S7. Obvious significant correlations between the microbiota and SCFAs and growth traits were found.

### Comparisons of predicted microbial pathways among divergent RFI and propionate groups

**Fig. 4:**
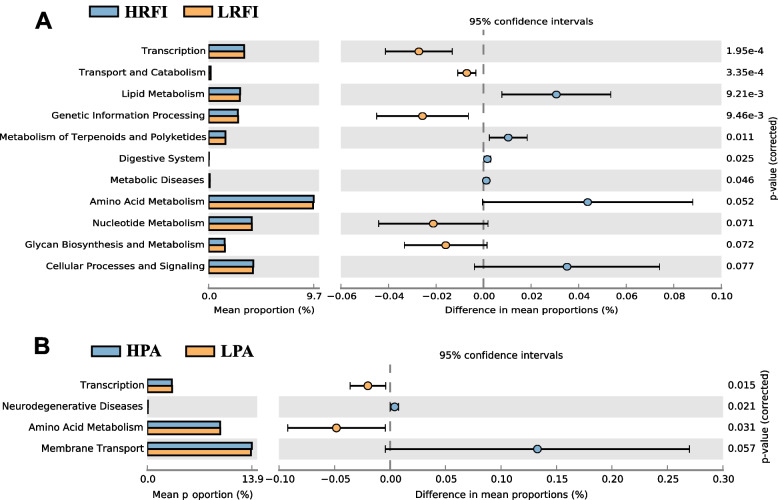
KEGG pathway prediction by the Greengenes database. **A** Comparison between divergent RFI groups. **B** Comparison between divergent propionate groups

Microbial KEGG pathways were predicted through the Greengenes database through PICRUSt. For divergent RFI groups (Fig. 4A), the enriched differential metabolic pathways included amino acid metabolism, lipid metabolism, nucleotide metabolism, glycan biosynthesis and metabolism, and informative pathways included transcription, genetic information process, and cellular processes and signaling. Groups with divergent propionate concentrations were used to conduct KEGG prediction as propionate had the highest correlation with RFI. The top 45 individuals in terms of propionate abundance were divided into the high group (HPA, mean: 0.127), and the bottom 52 individuals were divided into the low propionate abundance group (LPA, mean: 0.046) (Additional file 10: Figure S8. A). Four types of pathways were significantly differentially enriched between the divergent PA groups (Fig. 4B), including transcription, neurodegenerative diseases, amino acid metabolism, and membrane transport. The common enriched pathways in these two groups were found to be transcription and amino acid metabolism. The microbiota between the RFI groups could cause a difference in SCFA concentration.

### SCFA-related genetic variations and their effects on gut microbiota

**Fig. 5:**
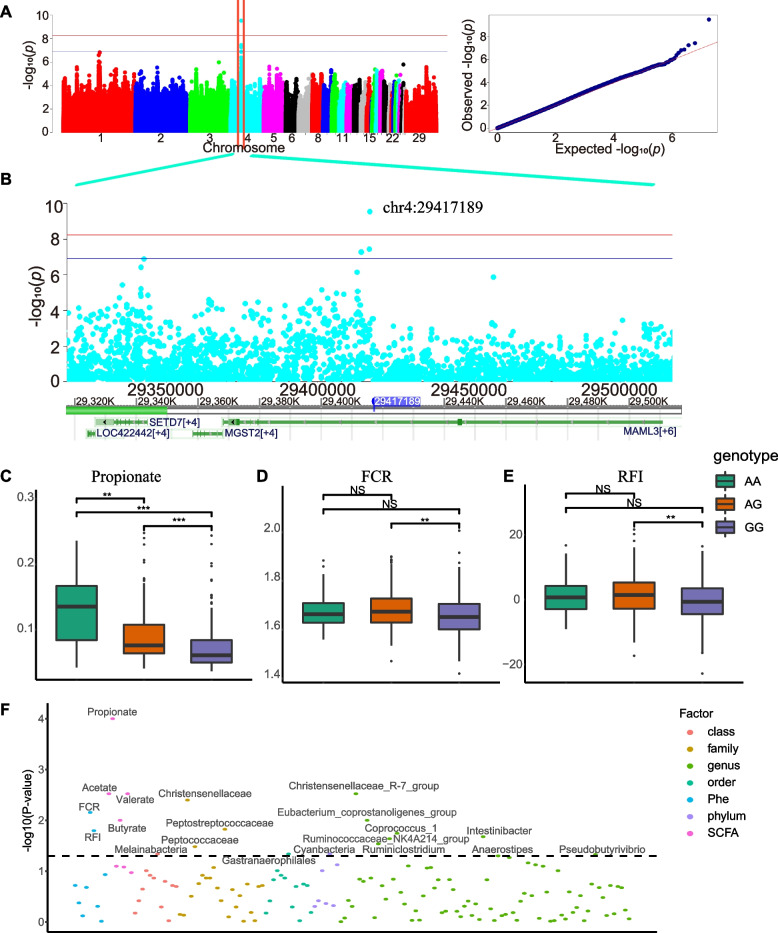
GWAS for propionate and associated SNP effects on RFI and the microbiota. **A** The Manhattan and QQ plots of propionate. **B** The significant region on chromosome 4 and gene distribution. **C**–**E** The effect of loci genotyping on propionate, FCR and RFI, ***, **, and ns represent adjusted P values < 0.001, < 0.01, and > 0.05, respectively. F Overview of the effect of the locus on grow performance, SCFAs, and the microbiota

The results in Fig. 3 show that there were correlations between SCFAs and growth traits and gut microbiota. Propionate and butyrate were positively correlated with RFI but negatively correlated with BRW and RBR. Propionate was positively correlated with ADFI. *Christensenellaceae* was negatively correlated with propionate, and *Christensenellaceae* and *Bacillaceae* were negatively correlated with butyrate. Thus, the association between the host genome and SCFA was examined through GWAS. Only butyrate, propionate, valerate, and isovalerate were significantly associated with host variation. The Manhattan and QQ plots of propionate illustrate the correlation between feed efficiency and the significant signals found on the host genome (Fig. 5A). Furthermore, the Manhattan and QQ plots of the other three SCFAs can be found in Additional file 11: Figure S9. The GWAS results of growth traits, including bodyweight at d40, average daily feed intake, average daily gain, FCR, and RFI were presented in Additional file 12: Figure S10. Only the FCR and RFI had similar genetic regions on chromosome 13, and no common genetic basis was found between SCFAs and feed growth traits. The SNP-based heritability of four SCFAs ranged from 0.183 to 0.401, and the annotation from the GWAS for SCFAs is shown in Table 2. One locus (chr4: 29,414,391–29,417,189) associated with propionate showed significant signals. *MAML3*,* MGST2*, and *SETD7* were found in a 100-kb upstream and downstream region of the top SNP (Fig. 5B). *MAML3* was found to be involved in the Notch signaling pathway, *MGST2* participated in glutathione metabolism, metabolism of xenobioticsm drug metabolism and metabolic pathways, and *SETD7* played roles in lysine degradation, metabolic pathways, and the FoxO signaling pathway. Moreover, the top SNP found by GWAS of propionate explained approximately 8.43% of the phenotypic variance, and all of the suggestive significant SNPs were located in the intron regions of the genes (Additional file 13: Table S3). The variation in chr4: 29,417,189: G > A resulted from a base transversion. Birds with the major genotypes had lower propionate concentrations than those with the other two genotypes. The average propionate concentrations for the GG, AG, and AA genotypes were 0.074, 0.096, and 0.131 µg/mg, respectively (Fig. 5C). Meanwhile, chickens with the GG genotype had a better feed efficiency, with low RFI (− 0.630 vs. 1.467) and FCR (1.658 vs. 1.685), than those of the AG genotype. However, the feed efficiency of the AA genotype was not significantly different from that of the other two genotypes (Fig. 5D, E). To further investigate the combined effects of the genotypes on phenotypes, microbiota, and SCFAs, the differences were analyzed among the different genotypes using a Wilcoxon rank-sum test. In addition to propionate, acetate, butyrate, and valerate also showed differential concentrations among the different genotypes. The relative abundance of fourteen taxa, including one phylum, one class, one order, three families, and eight genera, differed significantly among different genotypes (Fig. 5F).

**Table 2:** Annotation of the GWAS results on SCFAs

Traits	SNP-h^2^	Chr	Position	Top SNP	Gene	Pathway
Propionate	0.183	4	29,414,391 - 29,417,189	Chr4:29,417,189:G > A	*MAML3*	Notch signaling pathway
					*MGST2* (50 k upstream)	Glutathione metabolism Metabolism of xenobiotics Drug metabolism Metabolic pathways
					*SETD7* (70 k upstream)	Lysine degradation Metabolic pathways FoxO signaling pathway
Butyrate	0.242	2	-	Chr2:11,072,248:C > T	NA	
		7	-	Chr7:1,661,848:G > A	lnc_RNA	
Valerate	0.389	6	28,682,597 - 28,802,237	Chr6:28,682,597:T > C	*TDRD1*	
		6		Chr6:28,792,315:G > A	*ABLIM1*	
		6		Chr6:28,721,051:A > G	*AFAP1L2*	
Isovalerate	0.401	6	28,682,597 - 28,802,237	Chr6:28,682,597:T > A	*TDRD1*	
		6		Chr6:28,721,051:A > G	*AFAP1L2*	

The column “SNP-h^2^” is the SNP-based heritability, the column “Chr” is the Gallus gallus chromosome, the column “Position” is the region of SNPs above the suggestive line, the column “Top SNP” is highest the *P* value SNPs in each gene

### Christensenellaceae_R-7_group was identified as the biomarker related to the host genome, feed efficiency and SCFAs

**Fig. 6:**
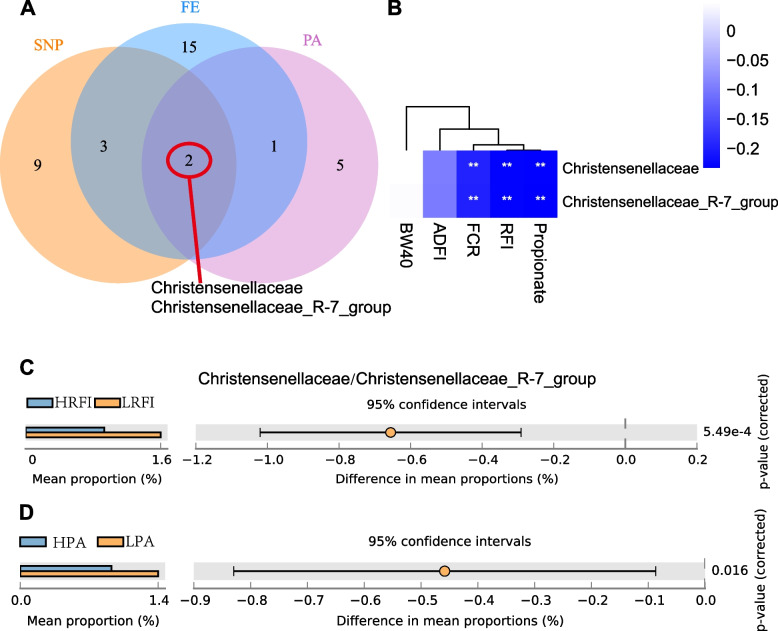
Biomarker determination from feed efficiency, propionate, and SNP genotyping effects. **A** The Venn diagram of selecting the biomarkers from SNP (locus genotypes), FE (feed efficiency), and PA (propionate) effects. **B** Spearman correlation between the biomarkers and different traits. **C**, **D** Comparisons of Christensenellaceae/Christensenellaceae_R-7_group among divergent RFI and PA groups, respectively

As described above, 14 taxa were associated with the top SNP associated with propionate. Two-part association and Spearman correlation analyses were used to identify the microbial taxa related to feed efficiency and propionate. The results of the two-part association model are presented in Additional file 14: Table S4. RFI and FCR were used to identify the representative taxa for feed efficiency traits, and 21 taxa were found (Additional file 15: Figure S11. A). Eight taxa were identified as associated with propionate using the same method (Additional file 15: Figure S11. B). The biomarkers were selected based on the intersection of the SNP-affected taxa and the taxa related to feed efficiency and propionate concentration. Two taxa, *Christensenellaceae* and *Christensenellaceae_R-7_group*, were identified as biomarkers related to host genome, feed efficiency, and propionate concentration (Fig. 6A). These taxa were also detected in the microbial composition detection among divergent RFI and PA groups (Fig. 2 & Additional file 10: Figure S8. D). Significant negative correlations were found with RFI, FCR, and propionate concentration. Slight negative correlations were found with BW40 and ADFI (Fig. 6B). By comparing the relative abundance of these two taxa, it was found that *Christensenellaceae_R-7_group* was the only genus found in *Christensenellaceae* family, because of the mean proportions of these two taxa were the same. The relative abundance of *Christensenellaceae_R-7_group* was approximately 1 ~ 2%, and higher relative abundance was found in the LRFI (Fig. 6C) and LPA group (Fig. 6D).

## Discussion

### The effect of RFI-related traits on the microbial community

Feed efficiency is a complex trait influenced by feed intake and body weight. Early feed efficiency studies reported correlations between the gut microbiota and gut microbial community [24, 28]. The lack of difference in α-diversity between the HRFI and LRFI groups here agrees with the results of previous studies in chickens [26], and similar results were also found in pigs [62]. Different groups from one population might show a similar α-diversity, and divergent RFI selection of pig lines indicated different α-diversity [63]. A difference in β-diversity was discovered in the divergent RFI groups, indicating that the specific microbiota affects RFI. However, different breeds and diets can influence the microbial composition, leading to different RFI-related microbiotas with different traits [15, 26, 64, 65]. A fecal microbiota transplant (FMT) trial that identified the fecal microbiota from chickens with high feeding efficiency could improve the feed efficiency in other chickens, and three microbial taxa (*Lactobacillus*,* Dorea*, and* Ruminococcus*) changed in abundance after chickens received this treatment [27]. Our results are consistent with Metzler’s result [27], one member of Ruminococcus (*Ruminococcaceae_UCG_014*) played an essential role in the low-RFI group, and two members of Ruminococcus (*Ruminococcaceae_UCG_008* and *Ruminococcaceae_UCG_009*) and Lactobacillus were enriched in high-RFI chickens. It was reported that a positive association between improved feed efficiency and the relative abundance of *Butyricicoccus* and *Faecalibacterium* is considered beneficial for the health of the animals [66]. However, our results did not agree with some previous studies, even in a white broiler population. In recent reports, *Oscillibacter* in the cecum and *Butyricicoccus* in the cloaca were more abundant in low-RFI chickens, and *Subdoligranulum variabile* in the ileum and two *Peptostreptoccaceae* members in the ileum and cloaca were negatively correlated with feed efficiency [26]. In our study, cecal *Butyricicoccus* presented a slightly positive correlation with RFI and nearly no correlation with SCFAs. These results were understandable because different GITs showed different microbial compositions so inconsistent correlations might have been observed. Meanwhile, *Subdoligranulum* showed a negative correlation with RFI, and a slightly negative correlation with butyrate, propionate, and acetate concentrations. These microbial taxa showed correlations with SCFAs, which could be a primary explanation for why propionate and butyrate concentrations were significantly correlated with RFI.

### SCFAs are representative metabolites of gut microbiota function in feed efficiency

Our study found that propionate and butyrate concentrations were significantly different between the HRFI and LRFI groups. Propionic acid is beneficial to the human body as it may play a role in satiety and energy homeostasis via specific mechanisms, including activation of free fatty acid receptors, reducing lipogenesis levels and glucose homeostasis [5]. Butyrate, the anionic part of dissociated butyric acid and its salts, has been implicated in various host physiological functions, including energy homeostasis, obesity, immune system regulation, cancer, and even brain function [67, 68]. Butyrate was reported to induce the relative mRNA expression of Mucin 2 and its secretion in goblet-like cells [69], as well as promote the assembly of occludin through the AMPK pathway [70].

*Christensenellaceae* and *Christensenellaceae_R-7_group* were the two taxa found to be biomarkers that showed correlations with propionate concentration, feed efficiency, and locus genotype effects. *Christensenellaceae* has been widely investigated in the human gut, which suggests that it is highly heritable, regulated by host genetics and inversely related to host body mass index (BMI) [71, 72]. High feed efficiency traits are usually accompanied by leanness and health performance, and it was reported that *Christensenellaceae* was more enriched in the leaner individuals [73]. Fecal propionate concentration was negatively correlated with feed efficiency [74]. A similar result was reported by Wang et al. [75], who showed that probiotics improved the feed efficiency, with decreased propionate concentration in the rumen. In a previous broiler study, butyrate supplementation improved feed efficiency without affecting the growth rate and decreasing abdominal fat deposition [76]. In this study, butyrate and propionate concentrations showed positive correlations with RFI, which indicated negative correlations with feed efficiency. One hypothesis is that a larger proportion of butyrate was transported into the blood. One literature reported that fermented butyrate and propionate in the caecum can be involved in quick utilization/absorption, and there are relatively high correlations between SCFAs in the cecum and those in the portal and aortic serum [77]. Some literature reported that blood butyrate concentrations could be associated with feed efficiency [78, 79]. An in vivo/vitro assay reported that approximately 90% of SCFAs were absorbed in the hindgut [80]. However, in our study, blood metabolites were not measured. The dynamic balance between the lumen concentration and blood concentration of butyrate could be a focus of future studies.

### The association between SCFA and host genome variants

Our study proved that seven cecal SCFAs were moderately to highly heritable and were the first to conduct the GWAS for caecal SCFAs. SNPs associated with propionate concentration were located near *MAML3*,* SETD7*, and *MGST2. MAML3*, a protein of the Mastermind-like proteins family, is a transcriptional coactivator of Notch signaling, and Notch signaling plays a pivotal role in development and homeostasis [81, 82]. *MAML3* was also associated with metastatic and WNT signaling activation [83]. Notch and WNT signaling pathways are critical components of the intestinal stem cell signaling network [84]. The weighted SNPs were annotated as *SETD7*, which is involved in lysine degradation in pigs with low feed efficiency [85]. Lysine intake plays an essential role in intestinal lysine transport and promotes feed intake associated with the piglet gut microbiome [86]. No reports have reported the correlation between feed efficiency and *MGST2*, but *MGST2* was not enriched in a range of metabolic pathways, indicating a potential effect on feed efficiency [87]. Similar regions and genes were found to be associated with valerate and isovalerate. *TDRD* is associated with spermatogenesis [88]. *AFAP1L2* is an adaptor activator of the PI3K-AKT pathway [89], and *ABLIM1* is involved in the PI3K/Akt/Rac1 pathway [90]. These results agreed with those of previous studies showing that SCFAs function as signaling molecules in several pathways [91]. In this study, the propionate-associated genomic variants showed a correlation with feed efficiency traits, indicating that the function of *MAML3*,* SETD7*, and *MGST2*, needs further investigation.

### The regulatory mechanism for the effect of SCFAs on feed efficiency

SCFAs are the major end products from the fermentation of gut microbiota. Propionate can be biosynthesized from the succinate, acrylate, and propanediol pathways by using succinate, lactate, and deoxyhexose sugar as substrates, respectively [92, 93]. Butyrate can be transformed from butyryl-CoA by phosphotransbutyrylase and butyrate kinase in a direct pathway and by the butyryl-CoA:acetate CoA-transferase route [94, 95]. In our study, the divergent RFI and PA groups had significant differential pathways in transcription and amino acid metabolism. Genes encoding SCFA-producing enzymes were found to be active in a range of microbial strains [96, 97]. Thus, enzyme activity could be a part of explaining the differential transcription levels observed in the present study. Pyruvate is involved in many energy metabolism pathways and is correlated with butyryl-CoA and acetate CoA metabolism, which are substrates for SCFA production. Moreover, pyruvate was reported to be a product of amino acid metabolism [98–100]. Thus, the difference in amino acid metabolism found in the RFI and PA divergent groups could be explained by the fact that pyruvate was produced by amino acid metabolism.

The effect of the top SNP (chr4: 29,417,189: G > A) from the propionate GWAS on feed efficiency traits and microbial relative abundance was evaluated in this study. From the phylum to genus level, 14 taxonomies showed significant differences in relative abundance. The different genotypes also resulted in different feed efficiency traits and caecum SCFA concentrations. The previous studies proved that animal dietary supplementation, which can improve growth performance and feed efficiency, was always along with increased SCFA in GIT [101–104]. However, no literature reported the causal effect between SCFA and feed efficiency. Meanwhile, a previous study reported that poultry caecal SCFAs were originally produced by microbiota [32]. In the selected 14 taxonomies, at the genus level, *Christensenellaceae_R-7_group* and *Pseudobutyrivibrio* were significantly negatively correlated with butyrate and propionate concentrations, while *Anaerostipes* and *Eubacterium_coprostanoligenes_group* were significantly positively correlated with butyrate or propionate concentrations. All four genera were reported belonging to the propionate or butyrate-produced microbial families [105–107]. However, the SCFAs could not be affected by only one of the microbiota variations and must be affected by the microbial composition evolutions in this selection group [106]. *Christensenellaceae_R-7_group and Eubacterium_coprostanoligenes_group* were both significantly correlated with ADFI, FCR, and RFI, but they had divergent effects. In this study, no supplementation was applied, meaning the maximum SCFA production is at a similar level. No literature reported the correlations between caecal SCFA and feed efficiency in a natural broiler population. Based on our previous hypothesis, a large proportion of SCFAs could be absorbed and unutilized in serum, and SCFAs do have an effect on feed efficiency traits [77]. The relationship between GIT, serum, and target organs needs to be investigated in further study. However, a pathway from host genome variants to feed efficiency can be illustrated. The different genome types could lead to varied microbial compositions, which cause differences in SCFA concentrations in the gut tract. Then, the SCFAs are absorbed into the blood and then utilized in organs, such as the brain, to control feed digestion and appetites, reflecting the changes in feed efficiency traits [5, 36, 37].

## Conclusion

Our study provides strong evidence of the pathway that host genome variants influence the cecal SCFA by influencing cecal microbiota and then regulating feed efficiency. Our study concluded that host genetic variation could regulate the caecal microbially derived SCFAs, which play a role in host feed efficiency-related traits. The SNP-based heritability results suggest that the SCFAs had moderate to high heritability (*h* ^2^ = 0.183 ~ 0.401). The GWAS showed that four out of seven SCFAs have significant associations with genome variants. SCAF concentrations, microbiota, and feed efficiencies were significantly different among different genotypes for the top SNP. Cecal *Christensenellaceae* and *Christensenellaceae_R-7_group* were identified as biomarkers contributing to the combined effect of host genetics and SCFAs on chicken feed efficiency.

### Supplementary Information

**Additional file 1:** **Table S1.** Feed ingredients and nutrients composition for chickens during the experiment.

**Additional file 2:** **Table S2.** Descriptive statistics for phenotypes.

**Additional file 3:** **Figure S1.** General description between growth performance.

**Additional file 4:** **Figure S2.** The LD decay of the whole genome.

**Additional file 5:** **Figure S3.** General description between SCFAs with the original data.

**Additional file 6:** **Figure S4.** General description between SCFAs with the log2-transferred data.

**Additional file 7:** **Figure S5.** Two-part association model description from Fu et al.

**Additional file 8:** **Figure S6.** Data description between high RFI and low RFI groups.

**Additional file 9:** **Figure S7.** Heatmaps of growth performance and SCFA with genera.

**Additional file 10:** **Figure S8.** Microbiota composition between high PA and low PA groups.

**Additional file 11:** **Figure S9.** Manhattan and QQ plots for other SCFAs.

**Additional file 12:** **Figure S10.** The GWAS results on growth traits.

**Additional file 13:** **Table S3.** Detailed information on the SNPs associated with the SCFAs.

**Additional file 14:** **Table S4.** Two-part model association on RFI, FCR and Propionate.

**Additional file 15:** **Figure S11.** Significant microbial taxa of divergent feed efficiency and propionate.

## Data Availability

The sequencing data generate in this study are available in the Genome Sequence Archive (GSA: https://ngdc.cncb.ac.cn/gsa/). The accession number of 16S RNA gene sequencing data is CRA005940. The accession number of resequencing data is CRA006625.

## Abbreviations

SCFA: Short-chain fatty acid
FE: Feed efficiency
FI: Feed intake
FCR: Feed conversion ratio
RFI: Residual feed intake
GWAS: Genome-wide association study
GIT: Gastrointestinal tract
MAF: Minor allele frequency
LD: Linkage disequilibrium
ASV: Amplicon sequence variants
GC–MS: Gas chromatography-mass spectrometry
LMM: Linear mixed model
PA: Propionate
FMT: Fecal microbiota transplant
BMI: Body mass index
