# Supplementary material for: Combined effect of microbially derived cecal SCFA and host genetics on feed efficiency in broiler chickens
Source: Microbiome. 2023 Sep 1;11:198. doi: 10.1186/s40168-023-01627-6 (PMC10472625; doi:10.1186/s40168-023-01627-6)
Supplement: Supplementary file 4 — Additional file 3: Figure S1. General description between growth performance. [file 40168_2023_1627_MOESM3_ESM.pdf]

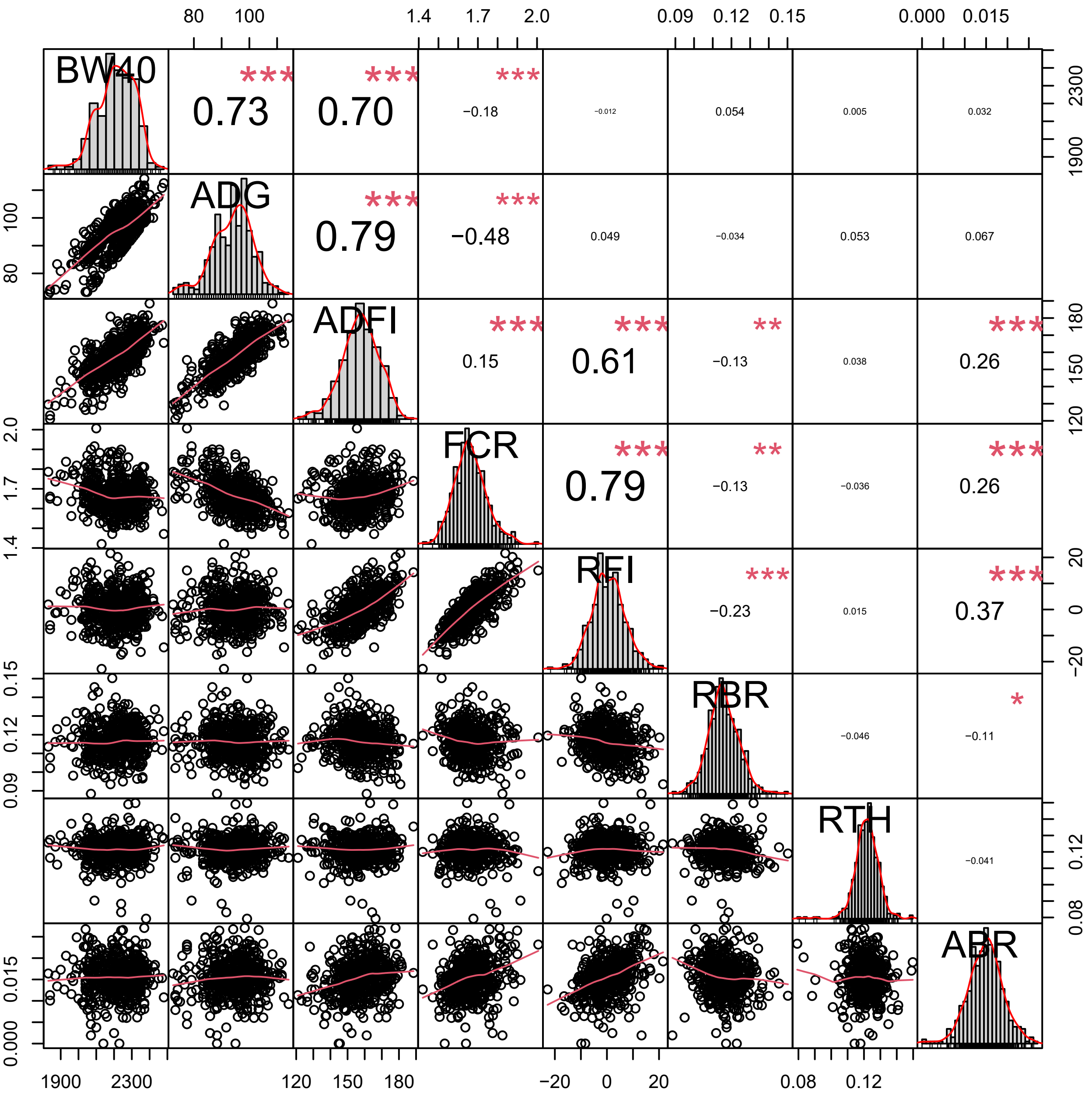

Figure S1. General description of growth performance. The diagonal is the data distribution charts. The upper triangle is the correlation index. The lower triangle is the scatter plots.

BW40 is body weight at day40; ADG is the average daily gain; ADFI is the average daily feed intake; RBR is the ratio of breast weight; RTH is the ratio of thigh weight; ABR is the abdominal fat ratio.
