# Supplementary figures and images for: Combined effect of microbially derived cecal SCFA and host genetics on feed efficiency in broiler chickens
Source: Microbiome. 2023 Sep 1;11:198. doi: 10.1186/s40168-023-01627-6 (PMC10472625; doi:10.1186/s40168-023-01627-6)

# LD decay

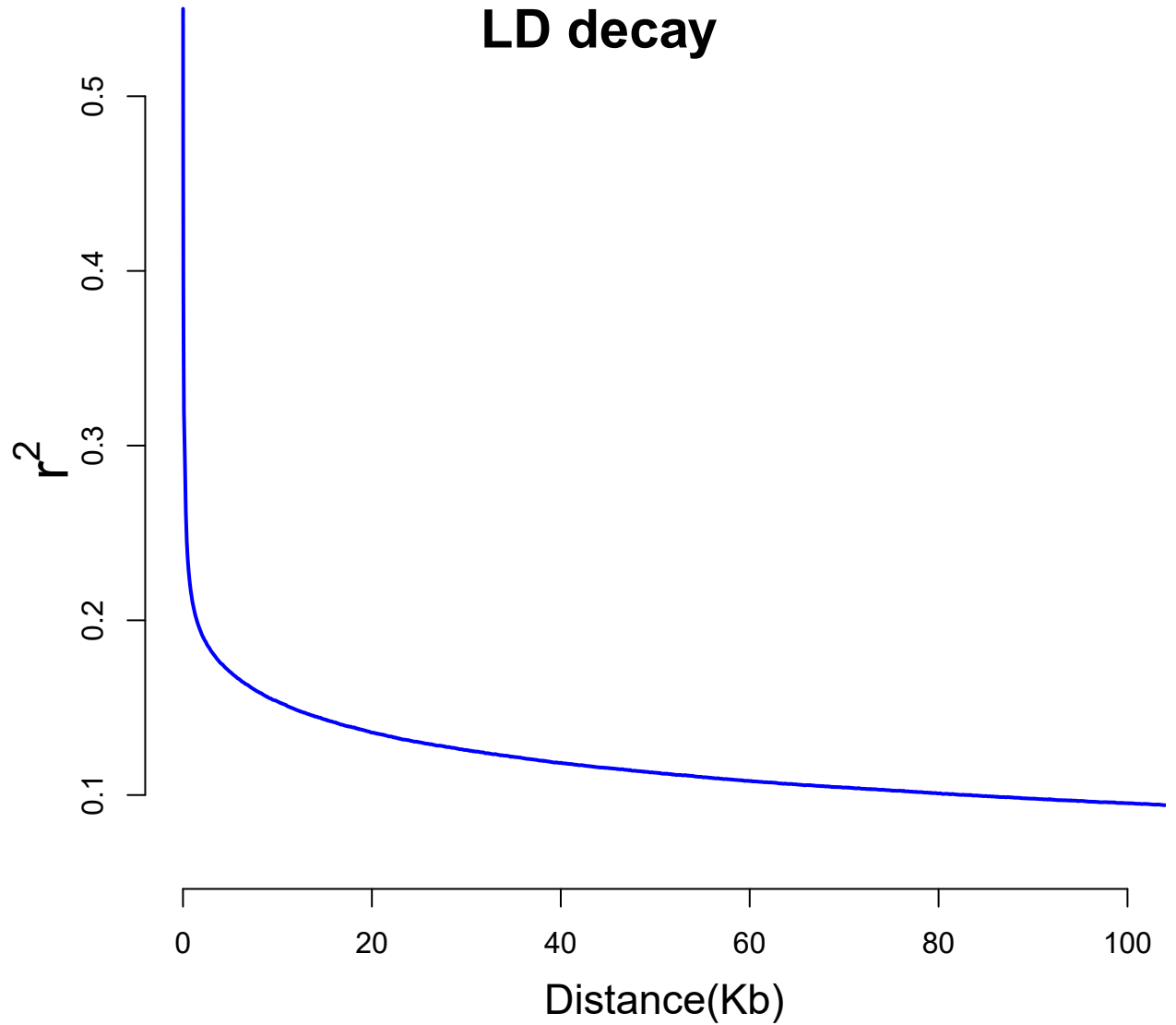

Figure S2. The LD decay of the whole genome.

Supplement: Supplementary file 5 — Additional file 4: Figure S2. The LD decay of the whole genome. [file 40168_2023_1627_MOESM4_ESM.pdf]

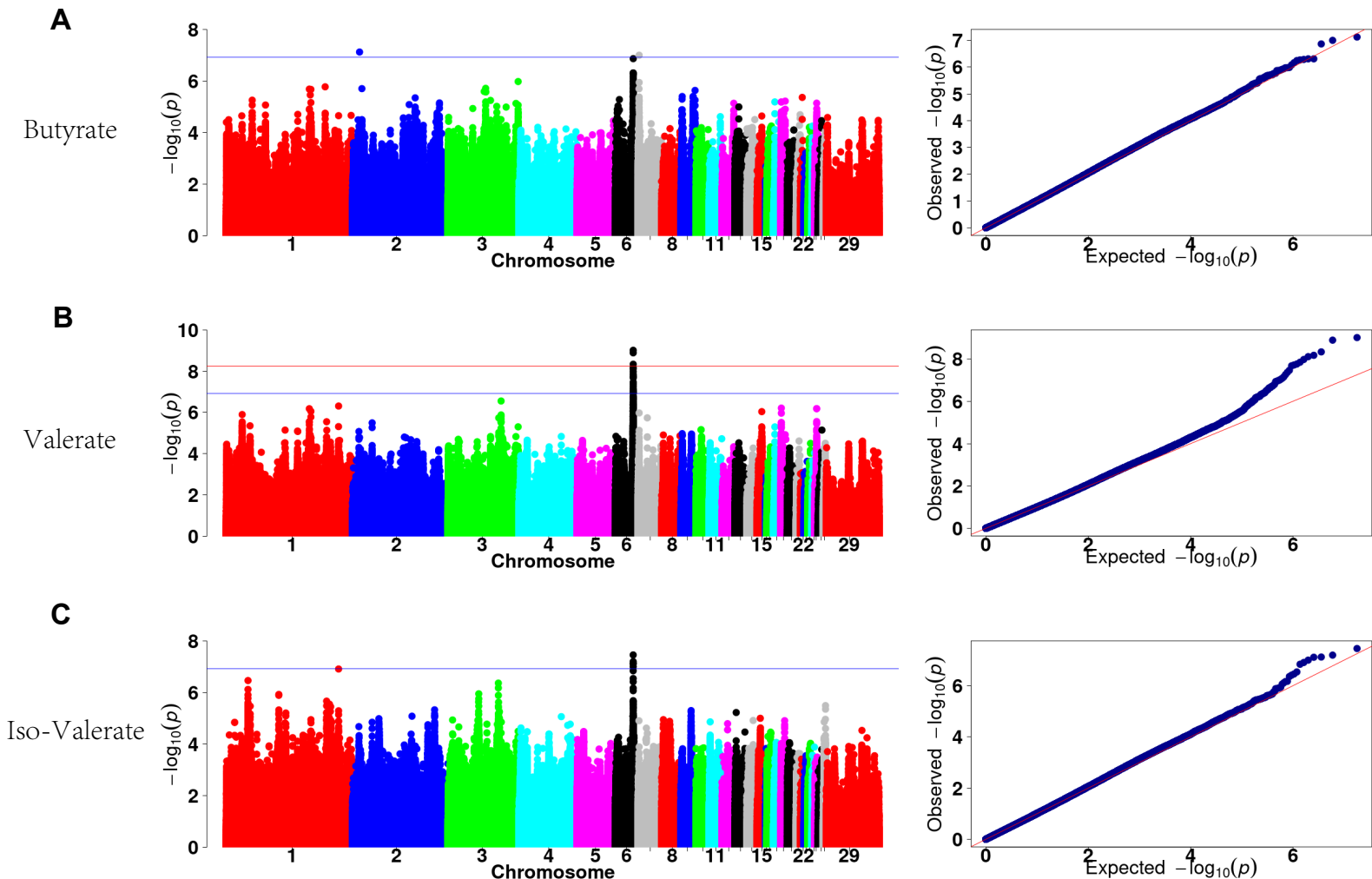

Figure S9. Manhattan and QQ plots for other SCFAs. A. Butyrate; B. Valerate,; C. Iso-Valerate.

Supplement: Supplementary file 12 — Additional file 11: Figure S9. Manhattan and QQ plots for other SCFAs. [file 40168_2023_1627_MOESM11_ESM.pdf]
