# Supplementary material for: Combined effect of microbially derived cecal SCFA and host genetics on feed efficiency in broiler chickens
Source: Microbiome. 2023 Sep 1;11:198. doi: 10.1186/s40168-023-01627-6 (PMC10472625; doi:10.1186/s40168-023-01627-6)
Supplement: Supplementary file 6 — Additional file 5: Figure S3. General description between SCFAs with the original data. [file 40168_2023_1627_MOESM5_ESM.pdf]

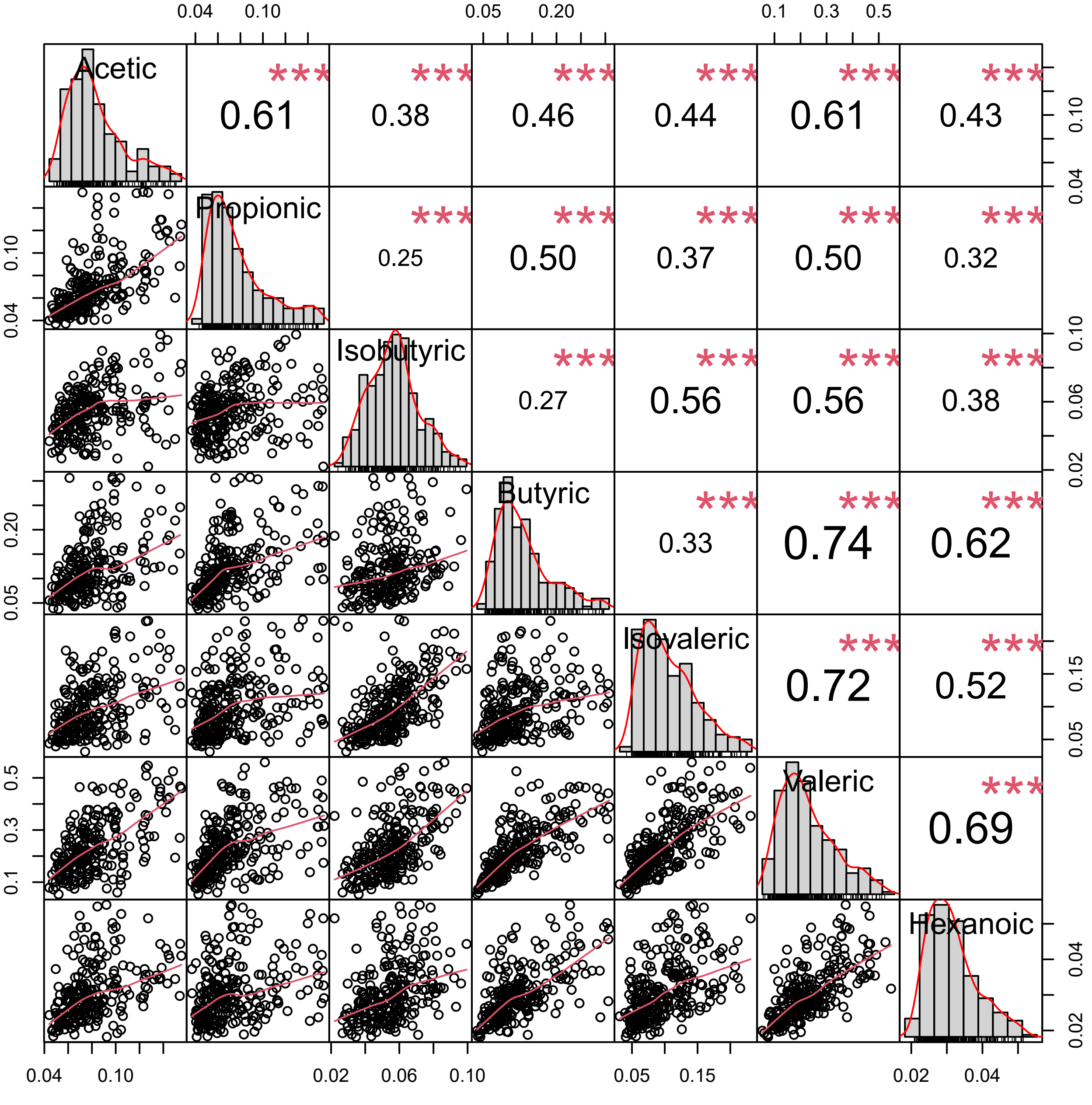

Figure S3. General description between SCFAs with the original data. The diagonal is the data distribution charts. The upper triangle is the correlation index. The lower triangle is the scatter plots.
