# Supplementary material for: Combined effect of microbially derived cecal SCFA and host genetics on feed efficiency in broiler chickens
Source: Microbiome. 2023 Sep 1;11:198. doi: 10.1186/s40168-023-01627-6 (PMC10472625; doi:10.1186/s40168-023-01627-6)
Supplement: Supplementary file 7 — Additional file 6: Figure S4. General description between SCFAs with the log2-transferred data. [file 40168_2023_1627_MOESM6_ESM.pdf]

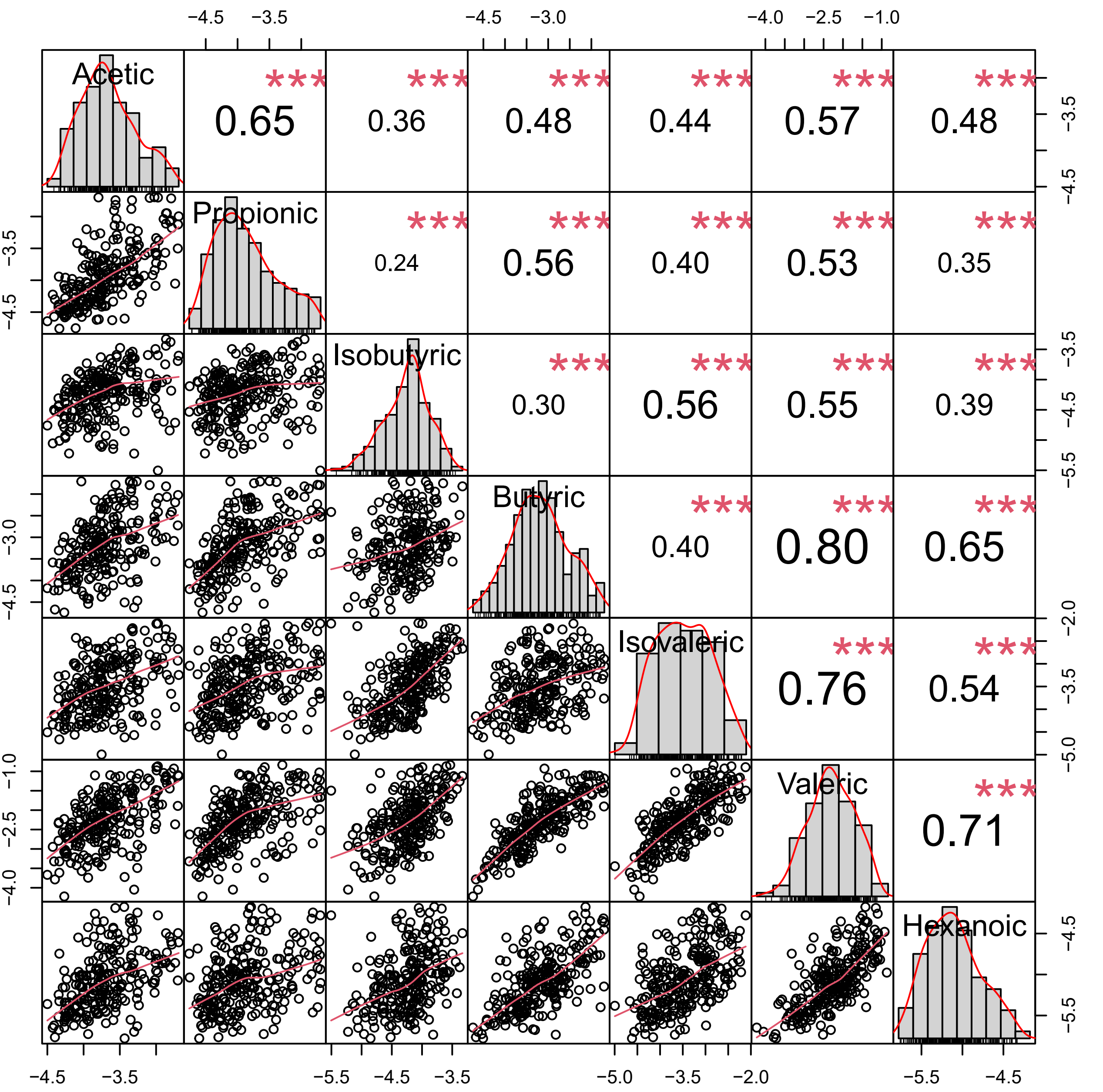

Figure S4. General description between SCFAs with the log2-transfered data. The diagonal is the data distribution charts. The upper triangle is the correlation index. The lower triangle is the scatter plots.
