# Supplementary material for: Combined effect of microbially derived cecal SCFA and host genetics on feed efficiency in broiler chickens
Source: Microbiome. 2023 Sep 1;11:198. doi: 10.1186/s40168-023-01627-6 (PMC10472625; doi:10.1186/s40168-023-01627-6)
Supplement: Supplementary file 8 — Additional file 7: Figure S5. Two-part association model description from Fu et al. [file 40168_2023_1627_MOESM7_ESM.pdf]

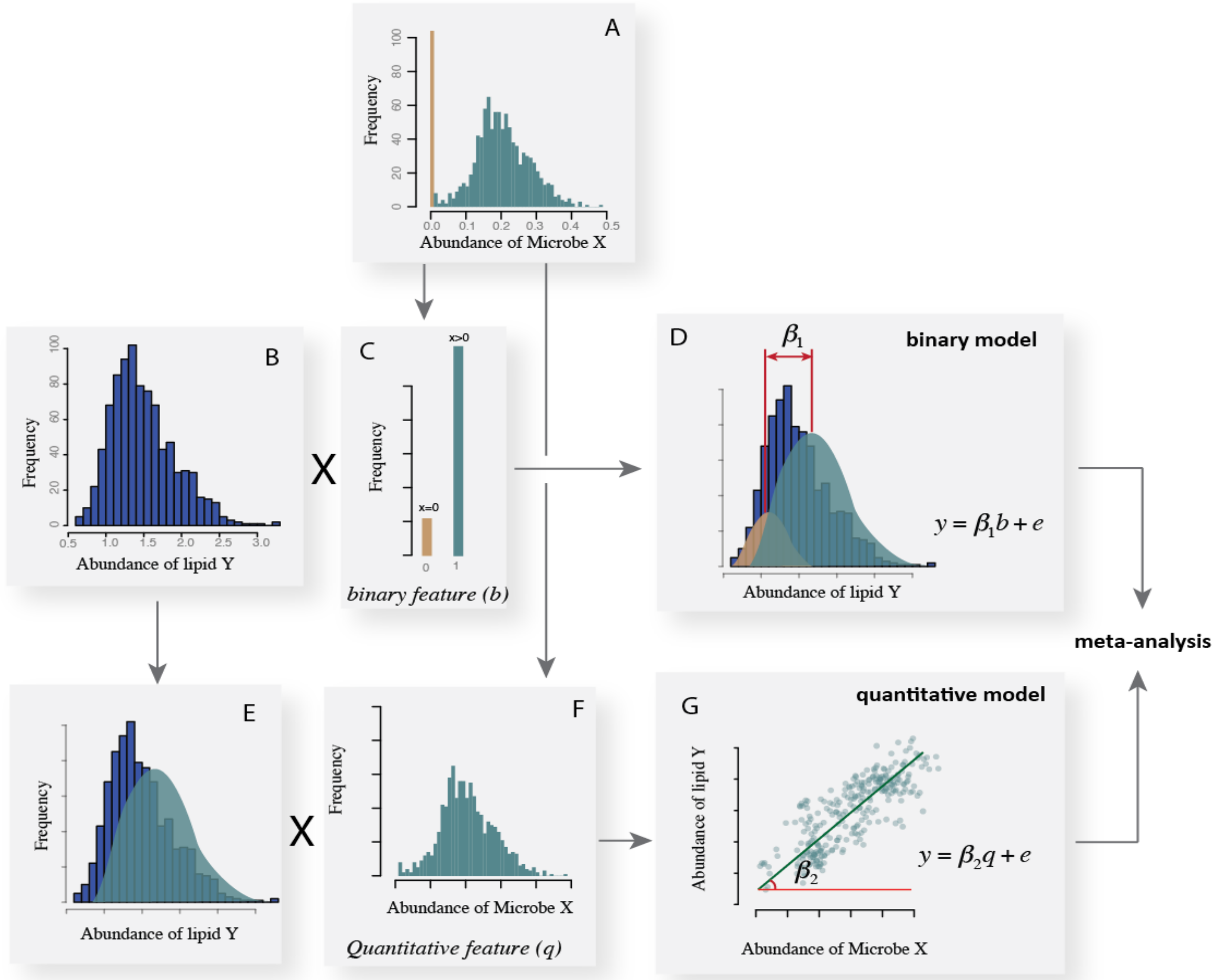

Figure S5. Two-part association model description from Fu et al. A. The distribution of microbe X. The brown bar indicates the number of subjects with zero value of microbe X, i.e. none of this particular microbe (also called "spike") and the green bars indicate the destruction of non-zero. The spike shows the departure of the distribution from a normal distribution. B. The distribution of lipid Y. After transformation, the lipid distribution is close to a normal distribution. C. The binary feature of microbe X. The subjects are divided into two groups (zero vs non-zero). D. The binary model to test whether the lipid level is significantly different between the two groups (zero vs non-zero). E. The distribution of lipid Y for the subjects with non-zero. F. Quantitative feature of microbe X: the distribution of non-zero values. G. The quantitative model to test whether the abundance of lipid X is associated with the abundance of microbe X. The signals from the binary model and quantitative model are combined using meta-analysis.
