# Supplementary material for: Combined effect of microbially derived cecal SCFA and host genetics on feed efficiency in broiler chickens
Source: Microbiome. 2023 Sep 1;11:198. doi: 10.1186/s40168-023-01627-6 (PMC10472625; doi:10.1186/s40168-023-01627-6)
Supplement: Supplementary file 9 — Additional file 8: Figure S6. Data description between high RFI and low RFI groups. [file 40168_2023_1627_MOESM8_ESM.pdf]

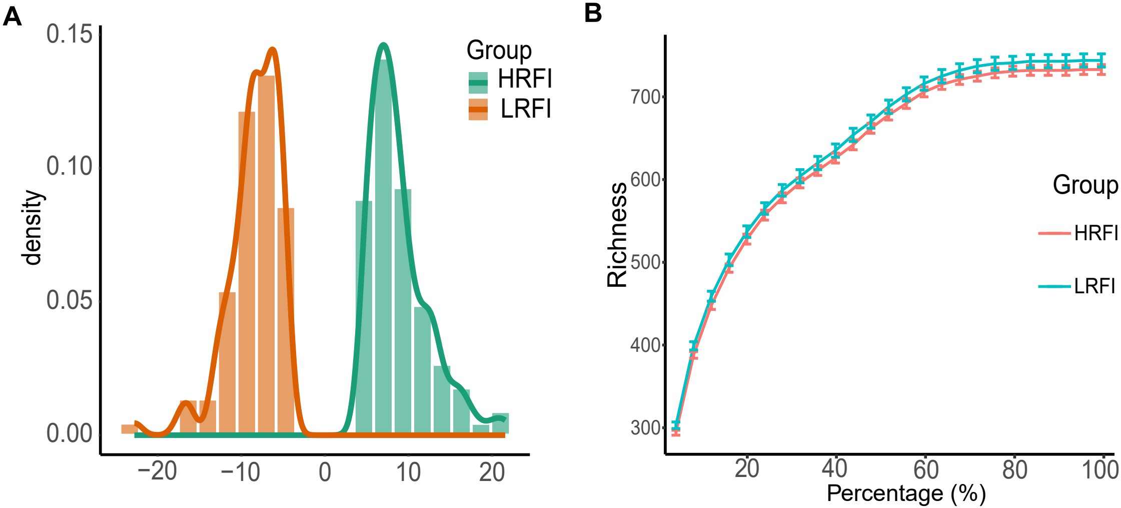

Figure S6. Data description between high RFI and low RFI groups. A. The RFI distribution of the high and low group. B. The rarefaction curve between different groups.
