# Supplementary material for: Combined effect of microbially derived cecal SCFA and host genetics on feed efficiency in broiler chickens
Source: Microbiome. 2023 Sep 1;11:198. doi: 10.1186/s40168-023-01627-6 (PMC10472625; doi:10.1186/s40168-023-01627-6)
Supplement: Supplementary file 10 — Additional file 9: Figure S7. Heatmaps of growth performance and SCFA with genera. [file 40168_2023_1627_MOESM9_ESM.pdf]

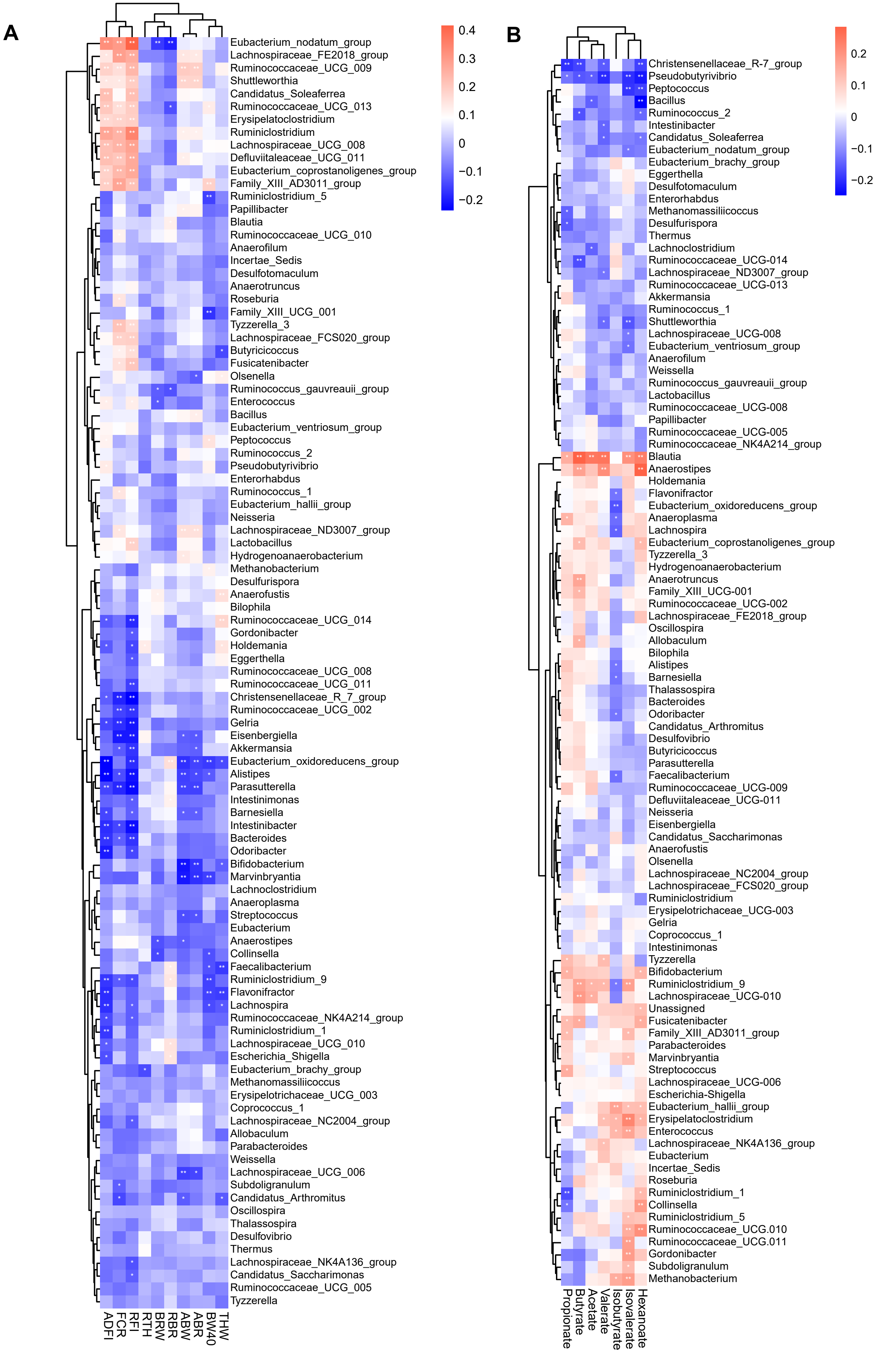

Figure S7. Heaptmaps of growth performance and SCFA with genera. A The correlation between growth traits and genera. B The correlation between SCFAs and genera.
