# Supplementary material for: Combined effect of microbially derived cecal SCFA and host genetics on feed efficiency in broiler chickens
Source: Microbiome. 2023 Sep 1;11:198. doi: 10.1186/s40168-023-01627-6 (PMC10472625; doi:10.1186/s40168-023-01627-6)
Supplement: Supplementary file 11 — Additional file 10: Figure S8. Microbiota composition between high PA and low PA groups. [file 40168_2023_1627_MOESM10_ESM.pdf]

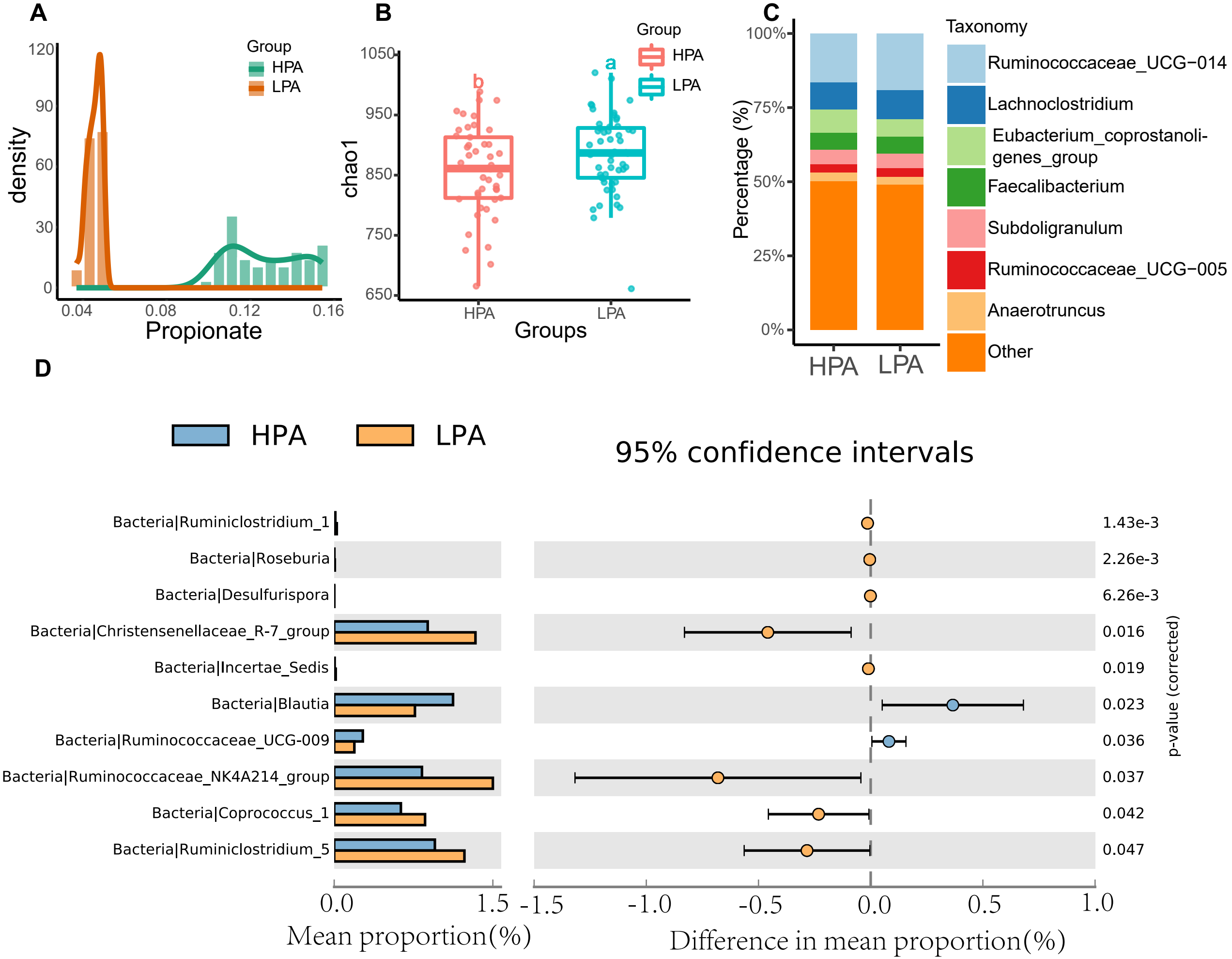

Figure S8. Microbiota composition between high PA and low PA groups. A. The RFI distribution of the divergent groups. B. The α-diversity index chao1 compared between two groups. C. The cecum microbiota composition of two groups on the genus level. D. The comparison of genus components between two groups.
