# Supplementary material for: Combined effect of microbially derived cecal SCFA and host genetics on feed efficiency in broiler chickens
Source: Microbiome. 2023 Sep 1;11:198. doi: 10.1186/s40168-023-01627-6 (PMC10472625; doi:10.1186/s40168-023-01627-6)
Supplement: Supplementary file 13 — Additional file 12: Figure S10. The GWAS results on growth traits. [file 40168_2023_1627_MOESM12_ESM.pdf]

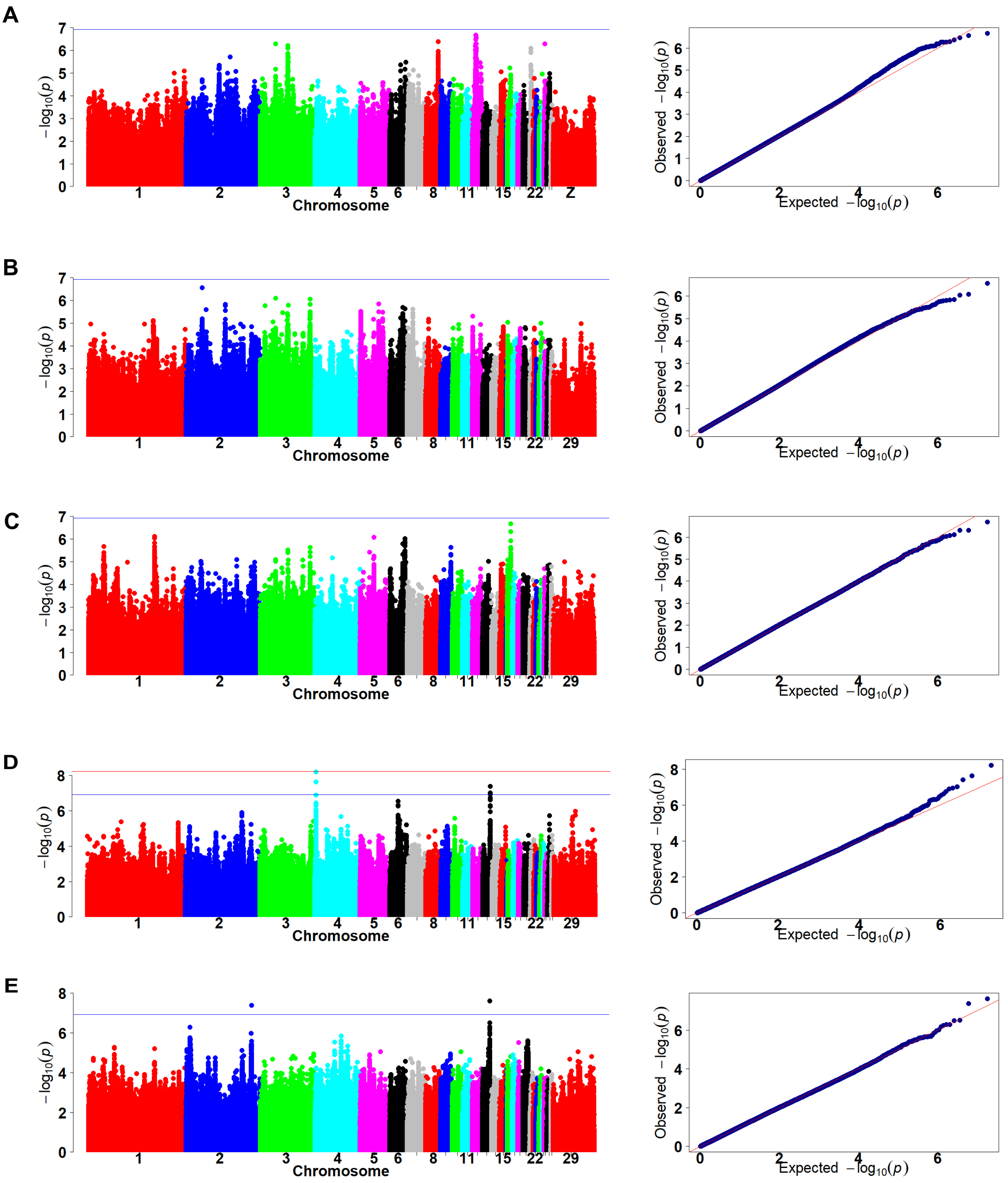

Figure S10. The GWAS results on growth traits. A the Manhattan and QQ plots of bodyweight at day 40. B the Manhattan and QQ plots of ADG. C the Manhattan and QQ plots of ADFI. D the Manhattan and QQ plots of FCR. E the Manhattan and QQ plots of RFI.
