# Supplementary material for: Combined effect of microbially derived cecal SCFA and host genetics on feed efficiency in broiler chickens
Source: Microbiome. 2023 Sep 1;11:198. doi: 10.1186/s40168-023-01627-6 (PMC10472625; doi:10.1186/s40168-023-01627-6)
Supplement: Supplementary file 16 — Additional file 15: Figure S11. Significant microbial taxa of divergent feed efficiency and propionate. [file 40168_2023_1627_MOESM15_ESM.pdf]

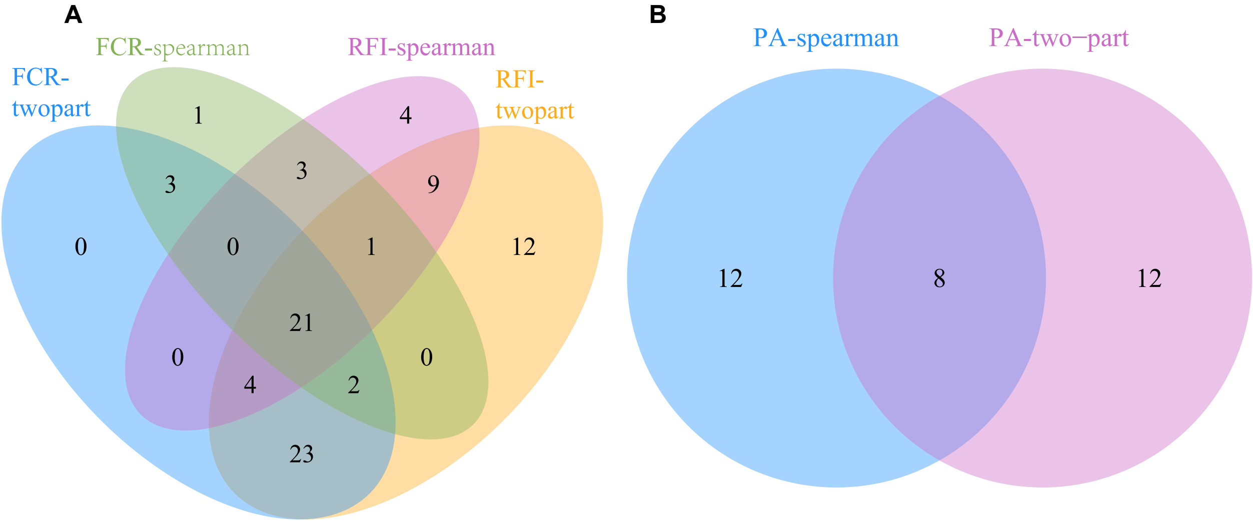

Figure S11. Significant microbial taxa of divergent feed efficiency and propionate groups. A. The Venn diagram of selecting the biomarkers from feed efficiency (FCR and RFI) . B. The Venn diagram of selecting the biomarkers from PA (propionate).
